# Changes in COVID-19 Perception and in TMD Prevalence after 1 Year of Pandemic in Italy

**DOI:** 10.1055/s-0042-1755192

**Published:** 2022-09-20

**Authors:** Giuseppe Scelza, Alessandra Amato, Roberto Rongo, Ludovica Nucci, Francesco D'Ambrosio, Stefano Martina

**Affiliations:** 1 Department of Medicine, Surgery and Dentistry “Scuola Medica Salernitana,” University of Salerno, Baronissi (SA), Italy; 2 Department of Neurosciences, Reproductive Sciences and Oral Sciences, University of Naples Federico II, Naples, Italy; 3 Multidisciplinary Department of Medical-Surgical and Dental Specialties, University of Campania Luigi Vanvitelli, Naples, Italy

**Keywords:** COVID-19 pandemic, psychological stress, dental practice, survey, temporomandibular disorders, TMD pain

## Abstract

**Objectives** The study aims to report the perception of dental practices and assess the individual's psychological stress level and related temporomandibular disorders (TMD) symptoms by evaluating the changes that occurred during the year following the first lockdown (May 2020).

**Materials and Methods** An online questionnaire was submitted to the Italian population between 20 March and 20 April 2021. It was sent via online platforms and included 14 questions.

**Statistical analysis** The data were analyzed using a chi-squared test and a comparison of the current data with the May 2020 survey data was performed using independent samples *t* -tests. The level of significance was set at *p* < 0.05.

**Results** Forty-three percent of subjects (872) considered the dental practice to be a place with a high risk of infection. Temporomandibular pain and joint sounds were reported by 35.7% (724) of the population; of these more frequently were women (71.8%, 520/724, *p* < 0.001). About 31% of the participants had moderate/severe psychological distress and, among them, it was found that 46.4% (294/634) reported TMD pain ( *p* < 0.001) and 34.9% (221/634) complained of temporomandibular joint sounds ( *p* < 0.001).

**Conclusions** Most participants did not consider dental practices as a place with a higher risk of coronavirus disease 2019 transmission and, in contrast to the data from a previous study, people were less afraid to go to dental practices. After the first year of the pandemic, the level of stress and depression increased and the prevalence of TMD pain and joint sounds increased, in association with gender, age, and level of distress.

## Introduction

Coronavirus disease 2019 (COVID-19) is a pandemic disease that broke out in China in December 2019 and quickly spread around the world. 1 The virus responsible for the disease was recognized and named severe acute respiratory syndrome coronavirus 2. 2

Dental practices are places at increased risk of COVID-19 infection due to both the proximity of the operator to the patient's mouth and contact with blood and body fluids, which is also exacerbated by high-speed rotating instruments. 3

In Italy, during the lockdown period, the COVID-19 pandemic negatively affected the professional activity; indeed dentists were afraid of contracting the virus and were aware of having to reduce the number of patients per day. 4 Moreover, a study conducted between March and April 2020 recorded a higher percentage of moderate/severe psychological stress in dental patients compared with the prepandemic period, and most of them considered the dental practice a high-risk place and were afraid to go to the dentist due to COVID-19. 5

At the end of December 2020, Italy's vaccination campaign against COVID-19 started but, due to the large number of vaccines to be produced, the time for administration was longer than expected, resulting in a concomitant rise in the number of infections (known as the “third wave”). 6 Thus, most of the population became discouraged about the resolution of the pandemic, in contrast to the first wave in which anxiety was accompanied by a feeling of a time-limited situation. For these reasons, in addition to the physical consequences of the disease, the COVID-19 pandemic also had a major impact on psychological aspects of individuals by increasing the risk of developing psychiatric and mental health disorders, such as depression, anxiety, and sleep disorders. 7 8 A systematic review investigating global prevalence and burden of depressive and anxiety disorders during the COVID-19 pandemic showed that two COVID-19 impact indicators were associated with increased prevalence of major depressive disorder and anxiety disorders. 9

Temporomandibular disorders (TMD) are conditions characterized by impaired function of the temporomandibular joint (TMJ), the masticatory muscles, and the neuromuscular system of the head and neck. 10 The prevalence of TMD is 10 to 15% in the general adult population, most commonly affecting patients between 20 and 40 years of age, women in particular, and approximately 27% in adolescents. 11 12

TMDs have a multifactorial etiology that mainly includes psychological conditions (anxiety and depression disorders, post-traumatic stress disorder), oral parafunctions, and traumatic factors. 13

Several studies have evaluated mental health using various measurement scores including the Patient Health Questionnaire 4 (PHQ-4), a scale that measures both the presence of anxiety and depression in an individual, finding a higher occurrence of psychological disorders during the pandemic. 7 14 15 However, there is no study that has quantified how much the indices changed over time during the pandemic.

Furthermore, the psychological distress during the COVID-19 pandemic might increase the risk of TMDs and oral parafunctions. 16 In Italy, a survey conducted in May 2020 and evaluated by means of PHQ-4 revealed an increased prevalence of self-reported TMDs related to pain during the lockdown compared with the prepandemic period, with 22.8% of respondents reporting TMD pain and 21.7% claiming high levels of distress. Interestingly, there was an association between high levels of distress and the presence of TMD pain after the onset of the pandemic. 5 However, no study has quantified the correlation between TMD and stress levels (PHQ-4) or has monitored the change in TMD prevalence and stress levels during more than 1 year of the pandemic.

Thus, the aim of our study is to investigate the effects of the continuing pandemic on patients' job activities, perceptions of the dental practice, and on the self-reported TMD symptoms and psychological distress. Findings will be compared with those of the previous survey performed at the end of the first Italian lockdown (May 2020).

## Materials and Methods

The study is a survey conducted by sending an online questionnaire to the Italian population in the period between March 20 and April 20, 2021. The study contains some similar questions to a survey 5 performed in May 2020 to compare whether there are any differences 1 year later. The questionnaire was generated on SurveyMonkey (SVMK, San Mateo, California, United States), an online survey development platform, and sent to participants via Facebook, WhatsApp, and email. Participants gave their consent for anonymous data processing and collection. The online questionnaire consisted of a total of 14 multiple-choice questions: 4 questions concerned personal information (age, gender, level of education, place of residence); 1 concerned the influence of COVID-19 on occupations; 2 were about the perceived risk of infection in the dental practice; 2 were about TMD screening (pain in jaw, temple, in the ear or in front of the ear and/or jaw joint sound(s) when opening/closing the mouth during the past year); and 4 questions from the PHQ-4. 17

The PHQ-4 is a four-item scale that assesses both anxiety and depression in individuals. It consists of 4 questions with a rating scale from 0 to 3 where 0 corresponds to “not at all” and 3 corresponds to “nearly every day.” A total score between 3 and 5 corresponded to mild stress, between 6 and 8 corresponded to moderate stress, and between 9 and 12 indicated severe stress. The PHQ-4 is divided into a 2-item PHQ-2, which focuses on depression, and the 2-item Generalized Anxiety Disorder (GAD-2), which focuses on anxiety. These data, therefore, can be analyzed independently.

### Statistical Analysis

Frequencies and percentages for categorical data were computed. A chi-square test was used to assess the association between gender (male vs. female) and level of distress. In case of a statistically significant association, logistic regression analyses to calculate the odds ratio (OR) were performed. Descriptive statistics also included mean and standard deviation of PHQ-4, PHQ-2, and GAD-2 values. An independent samples *t* -test was used to compare the means and standard deviations of PHQ-4, PHQ-2, and GAD-2 between data from a survey in May 2020 and present data between the TMD pain group and the general population. A standard statistical software package (SPSS, version 22.0; SPSS IBM, Armonk, New York, United States) was used. The level of significance was set at *p* < 0.05.

## Results

A total of 2027 (1217 F, 810 M) subjects completed the survey. The most common age groups of the participants were 18 to 29 years old (1103) and 30 to 39 years old (316). A total of 1721 patients lived with their families while 210 lived alone. Most patients had a high school (1001), undergraduate (706), or postgraduate (288) level of education ( Table 1 ).

**Table 1:** Descriptive statistics of population characteristics regarding age, gender, level of education, level of anxiety, and depression

Variable	Frequency (Percentage)						
Age	18–29 1103 (54.4%)	30–39 316 (15.6%)	40–49 158 (7.8%)	50–59 266 (13.1%)	60–69 136 (6.7%)	70–79 44 (2.2%)	80 or more 4 (0.2%)
Gender	Males 810 (40%)			Females 1217 (60%)			
Level of education	Primary school 6 (0.3%)	Secondary school 26 (1.3%)		High school 1001 (49.4%)	Undergraduate 706 (34.8%)	Postgraduate 288 (14.2%)	
PHQ-4	No/mild stress 1393 (68.7%)			Moderate/severe stress 634 (31.3%)			

Abbreviation: PHQ-4, Patient Health Questionnaire 4.

Almost 36% (721) of the participants stated that they had reduced or lost their job due to the COVID-19 pandemic. This data was associated with moderate/severe levels of stress reported with the PHQ-4 (OR = 1.7, *p* < 0.001). Indeed, 43.7% (277/634) of the participants claimed a reduction or loss of work ( *p* < 0.001).

Forty-three percent of subjects (872) considered dental practices to be places with a high risk of infection. Of these, 63.1% (550/872) were women, while men accounted for 36.9% (322/872), showing that women were more concerned about going to the dentist (OR = 1.2; *p* = 0.015). However, just over 63% of the participants (1284) had visited the dentist in the last year. Of these, 62% (796/1284) were women (OR = 1.2; *p* = 0.018) and 50.8% (652/1284) were young people (18–29 years; *p* = 0.001).

Regarding the items concerning TMD, 724 (35.7%) participants reported TMD pain, and 554 (27.5%) participants had perceived TMJ sounds during the past year. In both cases symptoms were more frequent in women (TMD pain 71.8%, *n* = 520/724, OR = 2.2; *p* < 0.001; TMJ sounds 66.4%, *n* = 368/554, OR = 1.5; *p* < 0.001). Furthermore, for both TMD pain and TMJ sounds, younger people (18–29 years) were the most affected ( *p* < 0.001).

Among the subjects with moderate/severe levels of stress, 71.8% (455/634) were women ( *p* < 0.001) and 64.7% (410/634) were young people (18–29 years; *p* < 0.001).

Furthermore, among individuals with moderate/severe stress, it was found that 46.4% (294/634) reported TMD pain ( *p* < 0.001) and 34.9% (221/634) complained of TMJ sounds ( *p* < 0.001) ( Table 2 ).

**Table 2:** Chi-squared test and regression analysis for loss of work, perceived risk, dental practice attendance, and TMD questions with gender and PHQ-4

	Gender		PHQ-4	
	Χ ^2^	OR (95% CI)	Χ ^2^	OR (95% CI)
COVID-19 caused a reduction or loss of work activity	0.577		<0.001	1.7 (1.4;2)
Dental practice considered to be a place at increased risk of COVID-19 infection	0.015	1.2 (1.1;1.5)	0.276	
Attended the dental practice in the last year	0.018	1.2 (1.1;1.5)	0.812	
Pain in your jaw, temple, in the ear or in front of the ear on either side during the past year (TMD pain)	<0.001	2.2 (1.8; 2.7)	<0.001	1.9 (1.6;2.3)
Jaw joint sound(s) when you opened or closed your jaw, during the past year (TMJ sounds)	<0.001	1.5 (1.2;1.8)	<0.001	2.1 (1.7;2.6)
PHQ-4	<0.001	2.1 (1.7;2.6)	−	−

Abbreviations: COVID-19, coronavirus disease; 95% CI, 95% confidence interval of the odds ratio; OR, odds ratio of the logistic regression model; Χ2, *p* -Value of the chi-squared test; PHQ-4, Patient Health Questionnaire 4; TMD, temporomandibular disorders; TMJ, temporomandibular joint.

The comparisons between PHQ data from the 2021 and 2020 surveys showed a statistically significant increase in Group 2021 compared with Group 2020 in all the assessed variables (PHQ-4 2021 = 4.68 ± 2.83 vs. PHQ-4 2020 was 3.96 ± 2.4, *p* < 0.001; GAD-2 2021 = 2.45 ± 1.59 vs. GAD-2 2020 = 2.21 ± 1.41, *p* < 0.001; PHQ-2 2021 = 2.25 ± 1.59 vs. PHQ-2 2020 = 1.85 ± 1.33, *p* < 0.001). Moreover, the comparison between patients with TMD pain and those without pain showed higher values of PHQ-4, GAD-2, and PHQ-2 in both TMD pain groups compared with the no-pain groups of the 2020 and 2021 surveys, and a statistical significant increase in the 2021 TMD pain group compared with 2020 TMD pain group ( Table 3 ).

**Table 3:** Means and standard deviations of PHQ-4, GAD-2, and PHQ-2 scores for both 2020 and 2021 samples

	Total			TMD pain			No pain			P (TMD pain vs. no pain)		
	PHQ-4	GAD-2	PHQ-2	PHQ-4	GAD-2	PHQ-2	PHQ-4	GAD-2	PHQ-2	PHQ-4	GAD-2	PHQ-2
2020	3.96 ± 2.4	2.21 ± 1.4	1.85 ± 1.3	4.35 ± 2.3	2.32 ± 1.4	2.02 ± 1.3	3.85 ± 2.4	2.06 ± 1.4	1.79 ± 1.3	0.001	0.001	0.005
2021	4.68 ± 2.8	2.45 ± 1.6	2.25 ± 1.6	5.47 ± 2.7	2.85 ± 1.5	2.62 ± 1.5	4.26 ± 2.8	2.22 ± 1.6	2.04 ± 1.5	<0.001	<0.001	<0.001
P (2020 vs. 2021)	<0.001	<0.001	<0.001	<0.001	<0.001	<0.001	<0.001	0.004	<0.001			

Abbreviations: GAD-2, 2-item Generalized Anxiety Disorder; PHQ-4, Patient Health Questionnaire 4; TMD, temporomandibular disorders.

Note: Independent two sample *t* -tests were performed to compare scores between years (2020 vs. 2021) and presence of TMD pain (TMD pain vs. no pain).

## Discussion

This study aimed to assess if there are any differences from the previous year in the patients' perceptions of dental practices, in patients' stress levels, and TMD symptoms due to the pandemic's persistence. This study is a continuation of a survey completed at the end of the first lockdown (May 2020). 5 The previous study was based on an online survey designed to assess perceptions of dental practices and various psychological aspects immediately after the end of the first lockdown. In 2020 study, 1,566 individuals answered the questionnaire. In the new survey, questions concerning the patient's perceived risk in the dental practice, the assessment of the degree of anxiety and depression, and the prevalence of TMD symptoms were in common with the old survey.

A total of 872 participants (43%) considered dental practices as a place of increased risk for COVID-19, showing a significant reduction compared with the previous year (55.3%). 5

Immediately after the end of the first lockdown, a previous study revealed dentists' concerns about returning to their practices due to the lack of personal protective equipment and the absence of vaccines. This feeling of apprehension also influenced patients, leading to lower dental practice attendance, except in emergencies. 18

Surprisingly, the higher perception of risk within the dental practice was recorded more frequently in younger (18–29 years) versus older patients, disagreeing with various studies. 5 19 The explanation for this could be that during the period in which the questionnaire data were collected the younger age groups in Italy had not yet had access to the vaccine and were therefore more worried about becoming infected than the elderly.

Despite the opinion that dental practice is risky, almost 64% of the participants (1284) went to the dentist during the last year. This may underline a greater confidence of patients compared with the previous survey in which only 57.1% stated that they would return to their dentist at the end of the lockdown. 5 This may depend on the dentist's more careful adherence to the recommended national guidelines for infection control and implementation of protective measures, such as appointment organization, waiting area organization, surface cleaning, hand hygiene procedures, and aerosol control (mask, visor, gown, rubber dam). 20

Two questions sought the presence of TMD during the last year. In this study, 35.7% of participants reported having TMD-related pain and 27.3% complained of TMJ sounds. These results show higher values than some previous studies. 5 14 21 In particular, Iodice et al 12 showed that in a sample of 4,299 Italian individuals, TMD pain and TMJ sound had a prevalence of 16.3 and 10.3%, respectively. Thus, it could be hypothesized that the COVID-19 pandemic led to an increased incidence of TMD symptoms in the Italian population.

The prevalence of TMD-related pain, which was 22.8% in our previous study, increased dramatically to 35.7% in just 1 year, showing the progressive onset of symptoms during the last year of the pandemic. Similarly, the prevalence of TMJ sounds increased from 19.4% in the previous year 5 to 27.3% in the latter study.

The results of our study are consistent with recent studies conducted in the last year that showed an increased prevalence of TMD in the population due to anxiety, depression, and worries during the COVID-19 pandemic. 22 23

Women showed TMD symptoms and TMJ sounds more frequently than men, reflecting findings and correlations found in the literature. 24 25

The PHQ-4 showed that 634 (31.3%) participants had moderate/severe psychological distress (score equal to or greater than 6). These results are significantly higher than those of the previous year, when only 21.7% of patients complained of these levels of psychological distress. 5 This showed that there was a progressive increase in distress during the last year, considering that 5% of patients reported moderate/severe values before the pandemic with the PHQ-4. 26 The comparisons of the data of the survey of 2021 with the survey of 2020 showed that the prevalence of people with moderate or severe levels of distress increased there was a statistically significant increase in the mean values for all the assessed variables. Several were the possible explanations that could have brought to the increase of the value of these variables: other studies found several distress sources that increase their weight in the daily life in the last year, such as the deterioration of relationships within the family, the maintenance of an unchanged daily routine, the change in the future economic situation, or the fear of personally getting COVID-19 or a family member getting infected and dying. 27 28 29

Other conditions involved in the worsening of the psychological aspect may be the increase in unemployment and the decrease in household income due to the pandemic. 17 In fact, our sample showed that 35.6% of the participants (721) had experienced a reduction or loss of their work activity and, among them, 38.4% (277/721) had moderate/severe stress levels.

Similar to the previous study, high levels of stress were found more prevalently in women and young people. 5 Furthermore, our data are consistent with various studies and in particular with a recent study by Beutel et al, which reports a higher prevalence of stress, anxiety, and loneliness in females and young patients. 30 31 32

In line with theories demonstrating the relation between stress and TMD, our study reports an association between increased PHQ-4 scores and TMD symptoms, 33 and in the 2021 survey there was an increase in the OR compared with the 2020 study (TMD pain OR 2021 = 1.9; TMD pain 2020 OR = 1.74; TMD sound OR 2021 = 2.1; TMD sound 2020 OR = 1.43). These findings highlight the worsening of TMD and distress symptoms possibly linked to the pandemic that have extremely change life of several people. 34

Considering the findings of this study, dentists should place great importance on the psychological aspect of the patient, which is crucial in the etiology and the treatment of TMD, 35 taking into account the clinical consequences that may arise from higher levels of anxiety, stress, and depression caused by the COVID-19 pandemic. In this situation, screening for and detecting possible signs and symptoms of depression and anxiety seems to be important in patients with TMD pain and, in some cases, it may be useful to seek the help of a specialist. 36 Further studies are needed to investigate whether this trend is continuing to grow over time.

This study has a limitation: it is a survey-based study, so the participants self-reported the information; moreover, it was not performed a sample size predetermination, considering that the objective was to achieve as more people possible involved in the study. However, it also presents some strengths, such as the high number of participants, the good representation of the population, and the possibility to compare the situation of dental patients in Italy 1 year after the start of the COVID-19 pandemic.

## Conclusions

Most of the participants did not consider dental office at greater risk of COVID-19 transmission and attended them in the last year, in contrast to the results of a similar study conducted at the beginning of the pandemic.

The level of anxiety, distress, and depression was higher after 1 year. TMD pain and sound in the population increased during the first year of the pandemic.
